# Non-Invasive measurement of the cerebral metabolic rate of oxygen using MRI in rodents

**DOI:** 10.12688/wellcomeopenres.16734.4

**Published:** 2022-08-25

**Authors:** Tobias C Wood, Diana Cash, Eilidh MacNicol, Camilla Simmons, Eugene Kim, David J Lythgoe, Fernando Zelaya, Federico Turkheimer

**Affiliations:** 1 Department of Neuroimaging, Institute of Psychiatry, Psychology & Neuroscience, King's College London, SE5 8AF, UK

**Keywords:** MRI, CMRO2, CBF, OEF, Glucose, Metabolism

## Abstract

Malfunctions of oxygen metabolism are suspected to play a key role in a number of neurological and psychiatric disorders, but this hypothesis cannot be properly investigated without an *in-vivo* non-invasive measurement of brain oxygen consumption. We present a new way to measure the Cerebral Metabolic Rate of Oxygen (CMRO _2_) by combining two existing magnetic resonance imaging techniques, namely arterial spin-labelling and oxygen extraction fraction mapping. This method was validated by imaging rats under different anaesthetic regimes and was strongly correlated to glucose consumption measured by autoradiography.

## Introduction

The brain requires around 20% of a human’s energy production, and hence requires a similar proportion of the body’s oxygen supply ^1, 2^. There is great interest in being able to quantitatively map the Cerebral Metabolic Rate of Oxygen (CMRO _2_) consumption, both as a marker of pathology and for the study of healthy ageing ^3– 6^. Although methods exist using oxygen isotopes with either Magnetic Resonance (MR) spectroscopic imaging or Positron Emission Tomography (PET) ^7– 9^, it would be advantageous to use proton-based Magnetic Resonance Imaging (MRI) methods due to their low invasiveness, lower cost, and wider availability. Recent years have seen the emergence of methods including whole-brain measurements of CMRO _2_ using a combination of T2-mapping and phase-contrast velocity measurements ^10, 11^, voxel-wise mapping using quantitative Blood Oxygenation Level Dependent (qBOLD) ^12^, BOLD calibrated with gas administration ^13, 14^ and high-resolution mapping methods based on Quantitative Susceptibility Mapping (QSM) ^12, 15^.

For this study we implemented a straightforward and robust method to measure CMRO _2_, which combines measurements of Cerebral Blood Flow (CBF) and Oxygen Extraction Fraction (OEF) made with a pre-clinical MRI scanner. We calculated CBF maps using Arterial Spin Labelling (ASL) ^16^. OEF maps were constructed by measuring the reversible rate of transverse relaxation R2′, which is related to the concentration of deoxyhaemoglobin (dHb) ^17– 19^.

We demonstrated our method by imaging rats with two anaesthetics known to affect brain metabolism differently, and compared these MRI measurements to gold-standard autoradiography measurements of glucose metabolism under the same anaesthetics. Although we found our MRI methods underestimated metabolism, we could still detect a relative effect between anesthetics.

## Methods

### Ethics statement

Study procedures were conducted in accordance with the Animal (Scientific Procedures) Act 1986 and with ethical approval from the King’s College London Animal Welfare And Ethical Review Body (AWERB) under the authorisation of license number P023CC39A. All harm to animals was prevented as procedures were performed under terminal anaesthesia. Animals were group housed under standard laboratory conditions with freely available food and water. There were no exclusion criteria for the animals.

### Theory

CMRO _2_, here measured in µmol/100g/min, is defined as the product of CBF, measured in ml/100g/min, and OEF multiplied by the constant *C _a_* which describes the amount of oxygen carried in arterial blood:

$$\;{\text{CMRO}}_{2}=\text{CBF}\times \text{OEF}\times C_{a}\;(1)$$

Throughout this paper we use a value of C _a_=8.48 µmol/ml, calculated from the values for mice given in Gagnon *et al.* ^20^. Typical values used for healthy humans are 8.04 and 8.33 µmol/ml ^13, 21^.

The measurement of CBF (measured in ml/100g/min) with ASL is a well-established MR method ^16, 22^. We chose to measure OEF from R2′, which is defined as the difference between the combined relaxation rate R _2_∗ and the irreversible relaxation rate *R*_2_ (R _2_∗ = *R*_2_ + R _2_′), where relaxation rates are the inverses of relaxation times (R _2_′ = 1/ T _2_′). MR images can be acquired with T _2_′-weighting using an Asymmetric Spin-Echo (ASE) sequence where the refocusing pulse is offset from the standard time to produce a spin echo, *T _E_*/2, by an echo-shift *τ*/2, which can be either positive (the pulse occurs later than *T _E_*/2 or negative (the pulse occurs earlier than *T _E_*/2 ^18^. Echoes formed at the same *T _E_* but different *τ* will hence have the same *T*_2_-weighting, but different amounts of additional T _2_′ (or R _2_′) weighting. By observing the signal in each voxel from multiple *τ* values, we can measure a mono-exponential R _2_′ as we would measure *R*_2_ from multiple values of *T _E_*.

However, in brain tissue the observed signal value at *τ* = 0 is less than would be expected from extrapolating the signal curve for *τ* ≠ 0 back to the origin. This discrepancy can be attributed to static dephasing of spins in susceptibility gradients. The principle biological contributor to such gradients is the presence of deoxyhaemoglobin (dHb) in capillaries and draining veins ^17^. In preference to the asymptotic equations used by Stone and Blockey ^18^ we adapt the full qBOLD equation from He and Yablonskiy ^23^:

$$S(\tau )=S_{0}exp(-DBV\times f_{c}(\text{δω}\times \tau ))\;(2)$$

where

$$f_{c}(\text{δω}\times \tau )=\frac{1}{3}\int_{0}^{1}du(2+u)\sqrt{1-u}\frac{1-J_{0}(1.5\text{δω}\times \tau \times u)}{u^{2}}$$

and δω=R _2_’/DBV is the characteristic frequency. We have neglected the dependence of *S*_0_ on TE and T _2_ for clarity. The OEF can then be found by

$$\mathrm{OEF}=\frac{3\text{δω}}{4\text{πγ}B0\text{δ}{\text{χ}}_{0}\mathrm{Hct}}$$

where *γ* =2π × 42.577 MHz is the proton gyro-magnetic ratio, B _0_ is the magnetic field strength, *δχ*_0_ = 0.264 × 10 ^−6^ is the susceptibility difference between oxygenated and deoxygenated blood cells, and we used a haematocrit (Hct) value of 0.34 ^19^. In previous clinical studies it has been possible to estimate DBV from the ASE data ^19^. We found that we could not reliably fit the data for both DBV and OEF at 9.4T and hence we fixed the value of DBV to 3.3% (see discussion) ^23^.

R2′ is not only affected by deoxygenated blood, but by any source of susceptibility gradients. The principal of these are background or Macroscopic Field Gradients (MFGs) from air/tissue interfaces, which can be corrected with Z-shimming ^18, 19^. A Z-shim is an additional small gradient played during the spin-echo formation which partially rephases signals in voxels affected by MFGs, but de-phases signal in unaffected voxels ^24, 25^. By acquiring and combining multiple images with different Z-shims, the lost signal from MFGs can be restored across the whole image, but will not affect the signal from sub-voxel susceptibility gradients due to deoxygenated blood ^19^. In the human brain the largest MFGs are present above the nasal sinus, where air is closest to the parenchyma, and hence the largest susceptibility gradient exists in the Z (axial, in humans superior-inferior) direction. In rodents, the largest voids within the head are the mastoids, and in addition the skull and the tissue surrounding the brain are significantly thinner than in humans. We hence found that gradients in the Y (in animals the superior-inferior) direction were also a significant issue and so added shimming in both the Z and Y directions.

### Imaging protocol

A total of ten adult male healthy Sprague-Dawley rats (440–537 g; Charles River) were imaged in a 9.4 Tesla pre-clinical MR system using a four-channel head receive coil, transmit body coil and separate ASL labelling coil (Bruker GmbH). All rats were initially anaesthetised by inhaling 5% isoflurane in an 80:20 mix of air and medical oxygen. Five of the rats were maintained with 2.5% isoflurane for the duration of scanning, while the remaining five received a bolus of 65 mg kg ^−1^ alpha-Chloralose ( *α*-Chloralose) solution in saline, administered through a tail vein cannula, followed by continuous infusion at a rate of 30 mg kg ^−1^h ^−1^.

All animals were scanned with the same protocol consisting of MP2-RAGE ^26^, ASL, and ASE images. The MP2-RAGE structural T1-weighted image was acquired with a matrix size of 160x160x128, isotropic 0.19mm voxel size, TE/TI1/TI2/TR = 2.7/900/3500/9000 ms, and flip-angles *α*_1_/ *α*_2_ = 7/9°. An additional Ultrashort Echo Time (UTE) COMPOSER scan was acquired for coil combination ^27^.

For ASL we used the manufacturer’s Continuous ASL (CASL) sequence with a spin-echo Echo Planar Imaging (EPI) readout ^22^. The matrix size was 96x96 with 18 axial (rostro-caudal) slices, 0.26x0.26x1.5 mm voxel size, TE/TR = 13.5/4000 ms, partial-fourier 66%, label time 3000 ms, post-label time 300 ms ^28, 29^, and 30 pairs of label/control images, scan time 4 minutes. The labelling plane was positioned 5 mm behind the carotid artery split, which was found using a localizer scan acquired with the labelling coil as per the manufacturer’s instructions. Two single-volume reference scans were acquired using the same sequence settings and no labelling power, one of which had reversed phase-encode direction (see below).

For the ASE sequence we modified the manufacturer’s spin-echo EPI sequence to allow the 180° refocusing pulse to be offset by *τ*/2 as defined above. The matrix size and resolution were matched to the ASL sequence, but with TE/TR = 70/1800 ms. Partial Fourier was switched off to minimise any intensity modulation from the echo moving out of the acquisition window in the readout (X, left-right) direction ^30^. Twelve values of *τ* spaced from -32 to 56 ms were acquired. At each, five Z-shims equally spaced from *G _Z_* = −0.8 to *G _Z_* = 0.8 mT m ^−1^ and nine Y-shims from *G _Y_* = −1.2 to *G _Y_* = 1.2 mT m ^−1^ were used. The Z-shim was incorporated into the slice-rephase gradient which lasted 2 ms and the Y-shim was played at the same time. The ASE scan lasted for 16 minutes and 12 seconds.

### Image processing and analysis

Image processing was carried out using a combination of FSL 5.0.1 ^31^, ANTs 2.1.0 ^32^ and QUIT 3.3 ^33^. Briefly, the complex MP2-RAGE structural images were first coil-combined ^27^ and then converted into both a T1 map and a uniform contrast image ^34^. From these, a study-specific template image was constructed ^35^ which was in turn registered to an atlas image ^36^. Eleven bilateral Regions Of Interest (ROIs) were selected from the atlas and transformed to the template space: the Thalamus (Thl), Hypothalamus (HThl), Striatum (Stri), Inferior Colliculus (InfC), Cingulate Cortex (CgCx), Retrosplenial Cortex (RtCx), Insular Cortex (InCx), Corpus Callosum (CC), Septum (Sptm), Dorsal Hippocampus (DHip) and Peri-Aqueductal Grey Matter (PAG).

The CASL images were corrected for motion ^37^ and susceptibility distortions ^38^, and then converted into a CBF map using the BASIL tool ^39^. The T1 of blood was set to 2.429 s ^40^, the labelling efficiency was set to 80%, and the distortion-corrected reference image was used as the proton density during CBF quantification ^41^. The reference image was registered to the MP2-RAGE structural image.

The ASE images with different Z- & Y-shims were first combined by taking the Root Sum-of-Squares (RSS) ^42^. To avoid noise amplification artefacts, we calculated the mean squared intensity in a background region and subtracted this from sum-of-squared images before taking the square root ^43^. The resulting shimmed ASE images were then motion and distortion corrected using the ASL reference data. The OEF was found from the corrected data by a non-linear fit to Equation 2 implemented in QUIT ^33^. We found that our images were too noisy to reliably fit for the parameter DBV, which is thought to be on the order of a few percent. To improve the quality of the fit for the remaining parameters we hence fixed DBV = 3.3% ^23^. We also observed that in certain brain regions the peak of our signal curve did not occur precisely at *τ* = 0, hence we introduced an additional parameter Δ *T* to account for this. The final free parameters were R2′, *S*_0_ & Δ *T*, from which the parameters *T*_c_, dHb and most importantly OEF could be derived. The resulting OEF and CBF maps were then multiplied together and by *C*_a_ to produce the CMRO _2_ map. The parameter maps were resampled into the template space and average ROI values extracted using the template-specific masks.

### Autoradiography protocol and analysis

To assess regional brain glucose metabolism we performed ^14^C-2-deoxyglucose (2DG) autoradiography, which measures Glucose Utilisation (GU) in µmol/100g/min as originally described by Sokoloff ^44^. We used a separate cohort of ten adult male Sprague Dawley rats (weight 325–380 g). All were initially anaesthetised for approximately 30 minutes with 2.5–3% isoflurane (in 80/20 medical air/oxygen), in order to cannulate their femoral and tail blood vessels for blood sampling and compound administration, respectively. After the cannulation, a local anaesthetic was applied and the wound sutured.

Isoflurane was then set to 2.5% for five rats. In the remaining rats, isoflurane was terminated and an intravenous bolus of 65 mg kg ^−1^ *α*-Chloralose was administered, followed by 30 mg kg ^−1^h ^−1^ infusion for the remainder of the experiment ^45^. Body temperature was maintained at 36 ± 0.5°C using a thermostatically controlled electric heating blanket and rectal probe.

Between 30 and 40 minutes was allowed for the rats to stabilise, after which we intravenously administered over 30 s 100 µCi/kg 2DG (Perkin Elmer, USA), and collected 14 timed arterial blood samples ^46^ over 45 minutes. After the final blood sample the animals were decapitated. Their brains were removed and frozen in −40°C isopentane and then stored at −80°C. Quantification of plasma glucose and ^14^C was carried out using a blood glucose analyser (YSI 2300) and scintillation counter (Beckman Coulter LS 6500), respectively. Brains were cryosectioned at 20 µm and exposed to X-ray film (Kodak Biomax MR-2) alongside calibrated ^14^C standards (GE Healthcare UK) for 7 days, after which they were developed in an automated X-ray film processor. Images were digitised using a Nikon single lens reflex camera and a macro lens, over a Northern Lights illuminator (InterFocus Ltd UK). Brain GU was calculated from the optical densities in the films using a calibration curve and the plasma glucose levels according to 44. We measured GU in eleven ROIs which matched those chosen from the MRI atlas, located at approximately +1, -3.5 and -8 mm from Bregma ^47^. Readings for each ROI were taken bilaterally from two or three adjacent brain sections and then averaged. The analyst was blinded to anaesthetic group.

### Statistical analysis

For statistical analyses we used the Python libraries pandas 1.0.5 and statsmodels 0.11.1 ^48^. The mean ROI values for each anaesthetic were compared with a non-parametric Mann-Whitney U-test with False Discovery Rate (FDR) multiple-comparisons correction. Finally, we compared our MRI oxygen metabolism measurements to the glucose metabolism measurements using a Robust Linear Model analysis of CMRO _2_ against GU. In this model, the slope of the line is the number of oxygen molecules consumed per molecule of glucose during metabolic activity, while the intercept gives the amount of oxygen consumed if no glucose was being consumed. Robust regression was used because residual variance was inhomogenous across the metabolic range. As our experimental design did not use the same animals for both CMRO _2_ and GU experiments, the measurements for each ROI were averaged across subjects (but not anaesthetics) before the regression, yielding a total of 22 data points for this analysis. For all analyses, a p-value of less than 0.05 was considered significant. ROI data and group average data are available in *Underlying data* ^49^.

## Results

### Pre-processing

**Figure 1.**
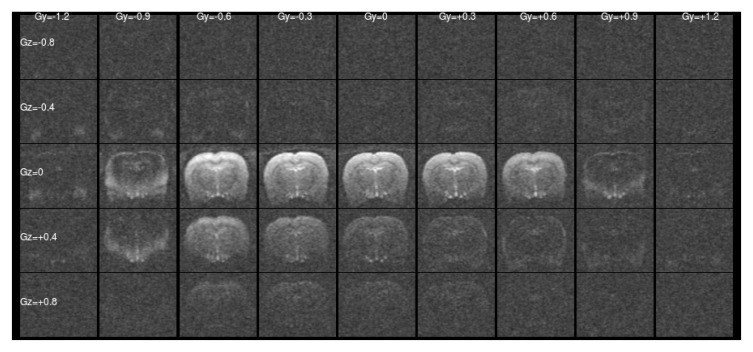
Raw asymmetric spin-echo data in a single slice at *τ* = 0 ms for all the values of Z- & Y-shims. The signal is concentrated at low shim values as expected.

**Figure 2.**
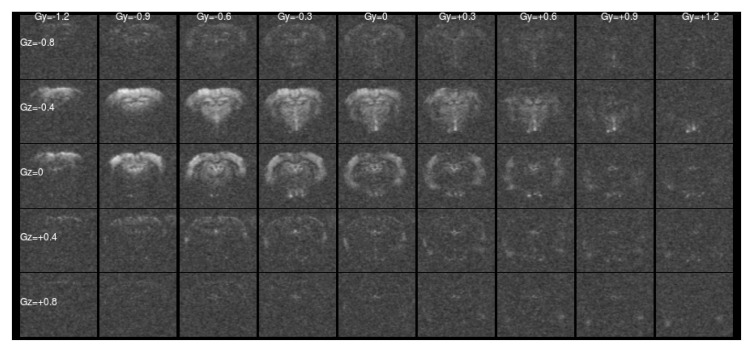
Asymmetric spin-echo data in a single slice at *τ* = 56 ms, as for Figure 1. For this highly asymmetric spin-echo, the signal energy has shifted towards more negative shim values, and the majority of the signal in the un-shimmed center image has been lost. Without shimming the signal would be erroneously low.

Figure 1 and Figure 2 show a single slice through all the raw ASE images collected with different values of Z-&Y-shims at *τ* = 0 and *τ* = 56 ms, respectively. The central images have both *G _Y_* and *G _Z_* equal to zero, i.e. in Figure 1 this is a simple unshimmed symmetric spin-echo image. In Figure 1 only the central, low value shims contain significant signal and the extreme shims are mostly noise, whereas in Figure 2 the unshimmed image is mostly noise and the signal has shifted towards negative values of *G _Y_* and *G _Z_*.

**Figure 3.**
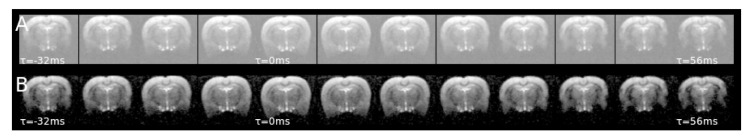
**A** The asymmetric spin-echo data after combining all shim values via naïve Root Sum-of-Squares. Noise has been amplified to the extent that the image cannot easily be distinguished from the background. **B** Noise suppression restores the signal-to-noise ratio to a reasonable level. The effect of R2′ decay can be observed at the high values of *τ* in cortical veins.

Figure 3 shows the result of combining all the different shim images via RSS both with and without noise suppression. Without suppression, amplification of the Rician noise is so severe that the background has almost the same intensity as the image. Subtracting the mean squared background intensity before the square-root operation restores the correct noise properties to the image, with crisp contrast between the image and background regions.

### Group comparisons

**Figure 4.**
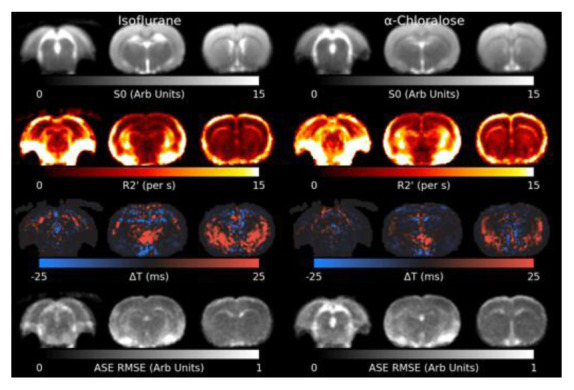
Slices through the fitted parameters and residual for the asymmetric spin-echo (ASE) data under both anaesthetics. R2′ values around the mastoid cavities are artifactually high. The model generally fits well across the brain, but is higher in white matter and cerebrospinal fluid. RMSE: Root Mean Square Error.

Figure 4 shows the results of the model fit to the shimmed ASE data. R2′ appears slightly higher in animals anaesthetised with *α*-Chloralose. Residual elevated R2′ can be observed surrounding the mastoid cavities and in a thin layer around the brain, where the Z-&Y-shimming was insufficient to correct extreme MFGs. The Root Mean Square Error (RMSE) is flat across most of the brain, indicating a reasonable model fit, but is elevated in white matter and cerebrospinal fluid (CSF), indicating the model fits less well in these areas. Δ *T* is increased towards the lower front of the brain.

**Figure 5.**
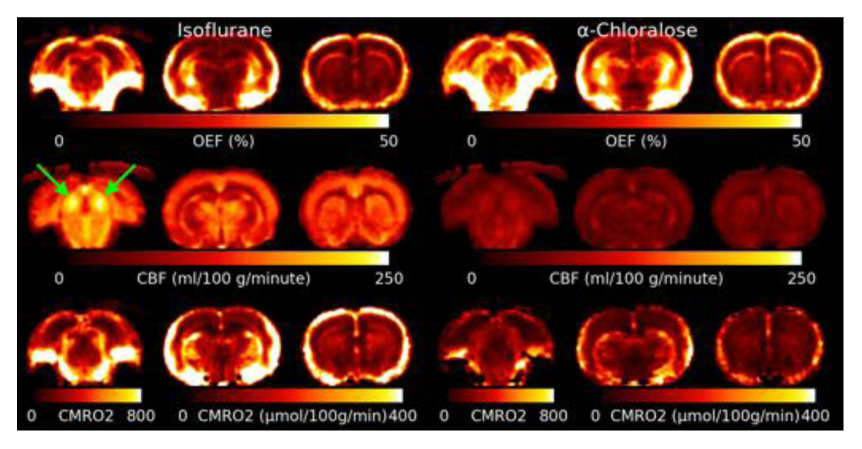
Slices through the mean Oxygen Extraction Fraction (OEF), Cerebral Blood Flow (CBF) and Cerebral Metabolic Rate of Oxygen (CMRO _2_) for both anaesthetics. CMRO _2_ is lower under *α*-Chloralose, however this is driven by a significant reduction in CBF as OEF is actually higher under *α*-Chloralose than isoflurane. Note that the slice through the inferior colliculus (marked with green arrows) for CMRO _2_ has a different color scale due to the much higher rate of metabolism compared to the other slices.

Figure 5 shows the mean OEF, CBF and CMRO _2_ for isoflurane and *α*-Chloralose anaesthetic. The OEF is higher under *α*-Chloralose. Areas with elevated R2′ due to MFGs also show artefactually high OEF. CBF is much lower under *α*-Chloralose anaesthetic than under isoflurane. The Inferior Colliculus shows an elevated CBF compared to other brain regions. CMRO _2_ is consistently higher under isoflurane than under *α*-Chloralose.

**Figure 6.**
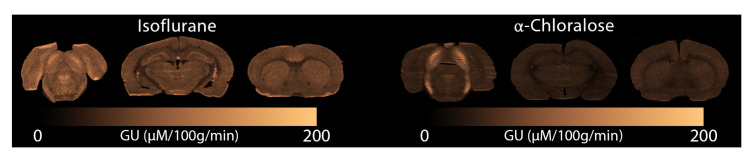
Glucose consumption measured with autoradiography under (left) isoflurane and (right) *α*-Chloralose. GU: Glucose Utilisation.

In Figure 6 we display glucose consumption under both anaesthetics. Similarly to the MRI data, glucose metabolism is clearly reduced under *α*-Chloralose compared to isoflurane, and the Inferior Colliculus displays elevated metabolism compared to the rest of the brain.

**Figure 7.**
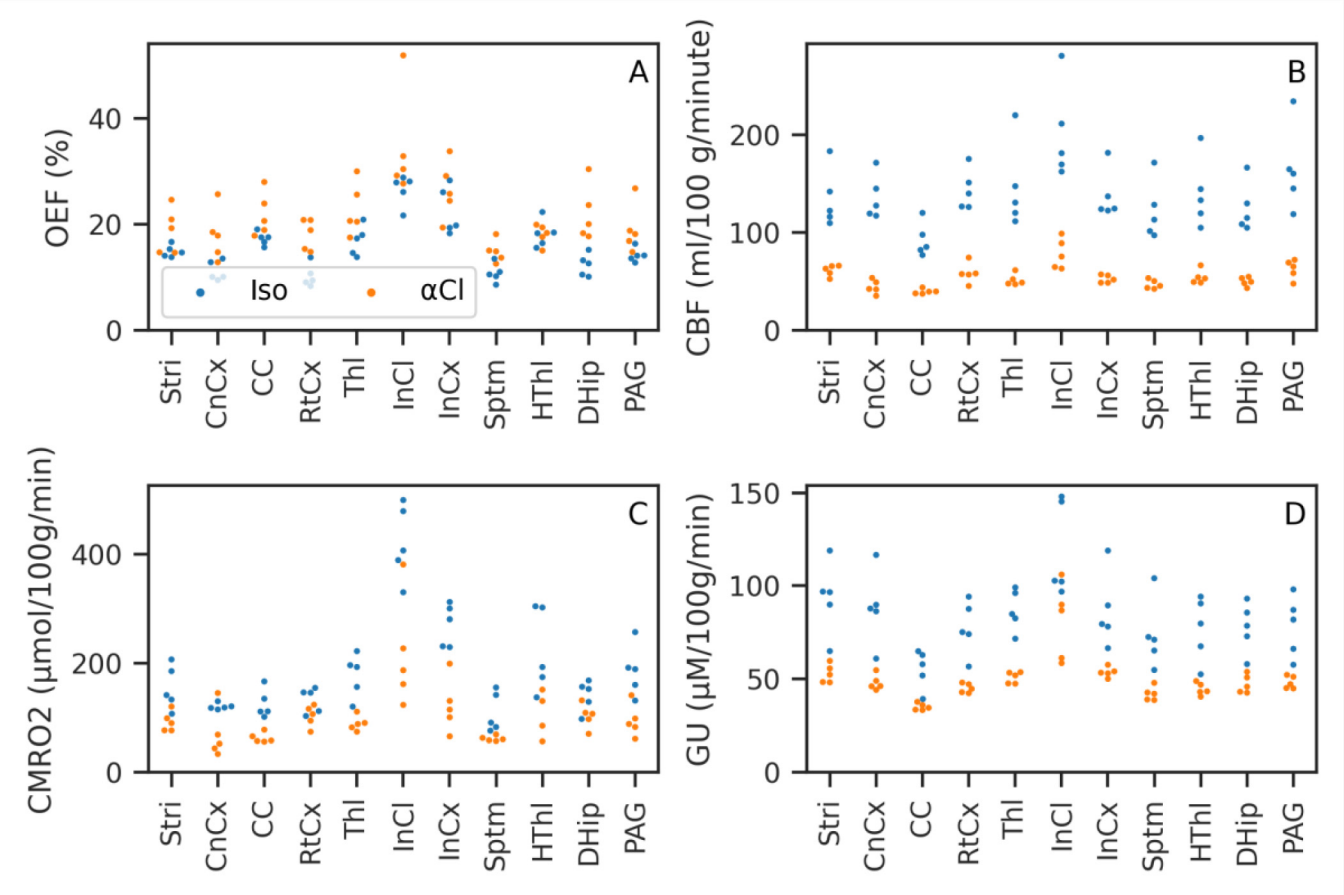
Mean value of Oxygen Extraction Fraction (OEF), Cerebral Blood Flow (CBF), Cerebral Metabolic Rate of Oxygen (CMRO _2_), and Glucose Utilisation (GU) in the chosen Regions of Interest (ROIs) for each subject. CMRO _2_ and GU consumption are both reduced under *α*-Chloralose anaesthetic compared to isoflurane. Almost total separation between the two groups was achieved; ROIs and parameters where this did not occur are noted in the text.

Table 1 gives the mean and standard deviation across subjects of each ROI for OEF, CBF, CMRO _2_ and GU. Figure 7 shows the same data plotted graphically. CMRO _2_, GU and CBF were all lower under *α*-Chloralose than isoflurane, while OEF was generally higher under *α*-Chloralose than isoflurane. These effects were strong and consistent for both CBF and Gu, with perfect separation between *α*-Chloralose and isoflurane, i.e. all values in one group higher/lower than the other, with the exception of the Inferior Colliculus glucose consumption (Mann-Whitney U=22, FDR corrected p=0.17). For OEF there was some overlap between the groups, in particular the Hypothalamus showed equal OEF (U=12, FDR corrected p=1). CMRO _2_ hence showed a smaller separation than GU or CBF, which despite large non-parametric test statistics did not survive multiple comparisons correction (majority of ROIs U>=24, uncorrected p≤0.017, FDR corrected p=0.07).

**Table 1.** Mean and standard deviation of each parameter value in each Regions of Interest (ROI), and the average across the ROIs. OEF, Oxygen Extraction Fraction; CMRO2, Cerebral Metabolic Rate of Oxygen; CBF, Cerebral Blood Flow; GU, Glucose Utlilisation.

ROI	OEF (%)		CBF (ml/100g/min)		CMRO _2_ (µmol/100g/min)		GU (µmol/100g/min)	
	Iso	αCl	Iso	αCl	Iso	αCl	Iso	αCl
Stri	14.9±1.1	18.9±1.1	134.8±29.8	61.3±29.8	155.1±40.6	92.7±40.6	93.5±19.3	52.9±19.3
CnCx	11.2±1.9	18.0±1.9	136.2±22.6	44.5±22.6	120.7±5.8	68.9±5.8	88.3±19.7	48.1±19.7
CC	17.3±1.2	21.9±1.2	92.6±17.2	39.7±17.2	125.4±26.2	63.2±26.2	55.4±10.3	35.1±10.3
RtCx	10.3±2.1	18.2±2.1	144.0±20.5	58.6±20.5	132.6±23.2	103.2±23.2	77.6±14.4	45.1±14.4
Thl	16.9±2.8	22.9±2.8	146.1±43.6	51.5±43.6	177.9±39.8	89.4±39.8	86.9±11.1	50.9±11.1
InCl	26.5±2.9	34.5±2.9	201.4±48.4	78.4±48.4	421.5±69.0	216.4±69.0	119.0±25.3	80.5±25.3
InCx	22.4±4.5	26.5±4.5	138.1±25.2	52.6±25.2	271.1±38.6	122.5±38.6	86.6±19.8	53.7±19.8
Sptm	10.8±1.8	14.9±1.8	122.5±30.1	47.1±30.1	109.6±36.5	61.8±36.5	73.6±18.4	42.1±18.4
HThl	18.2±2.6	18.1±2.6	139.9±35.2	54.5±35.2	222.6±76.7	80.9±76.7	76.9±17.1	44.6±17.1
DHip	12.3±2.1	22.0±2.1	125.0±25.1	49.9±25.1	141.1±28.1	103.3±28.1	77.7±13.3	47.3±13.3
PAG	14.2±1.3	19.1±1.3	164.8±42.9	62.7±42.9	186.2±46.9	94.6±46.9	78.2±16.2	48.2±16.2
Avg	15.9±5.3	21.3±5.3	140.5±38.8	54.6±38.8	187.6±95.6	99.7±95.6	83.1±21.5	49.9±21.5

**Figure 8.**
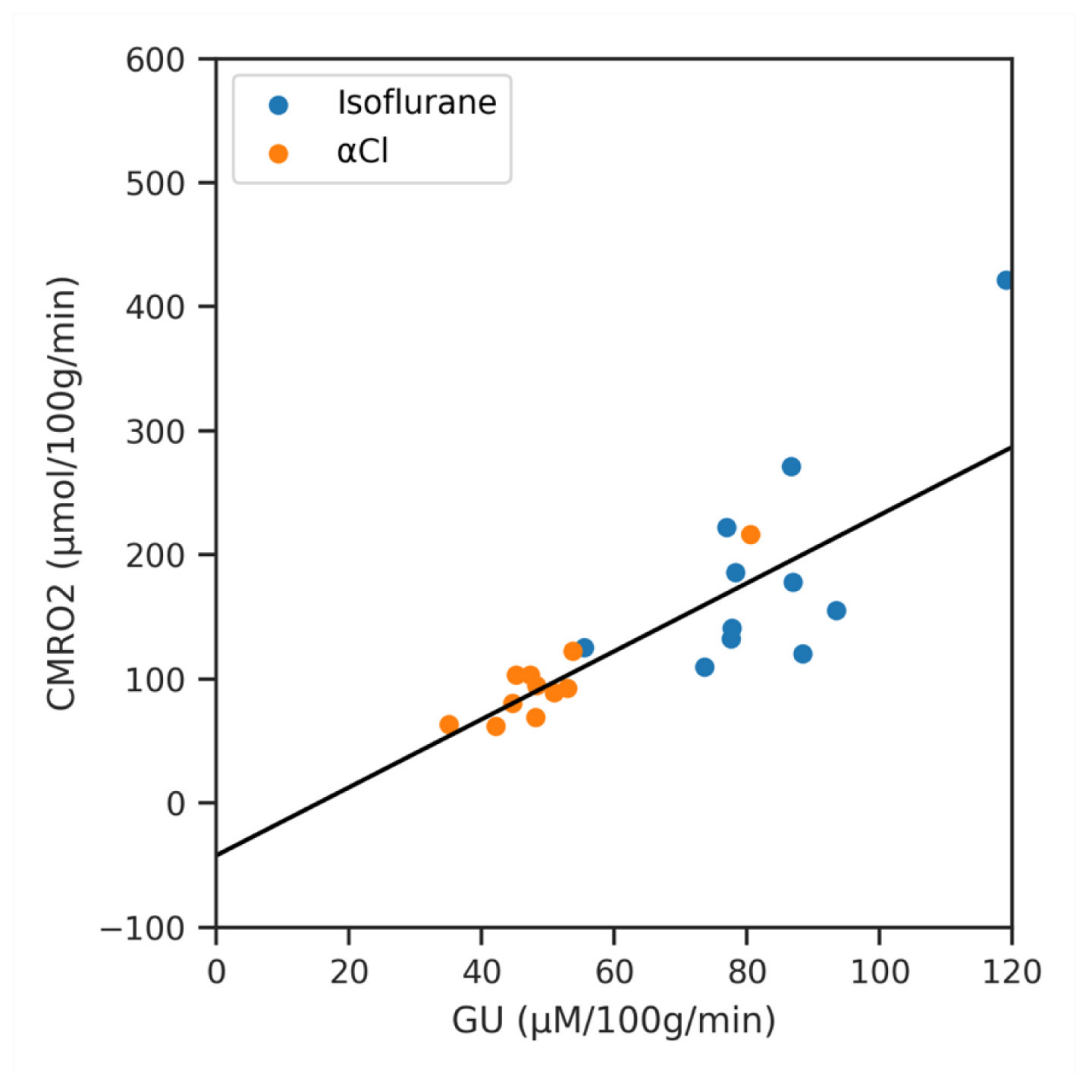
A regression analysis of Cerebral Metabolic Rate of Oxygen (CMRO _2_) against Glucose Utilisation (GU) across the averaged regions of interest data for both anaesthetics.

Finally we show the result of regressing CMRO _2_ against GU for the different regions of interest (averaged across subjects) in Figure 8. The slope of the line of best fit was 2.74 ( *p* < 0.001, 95% CI 1.96 to 3.53).

## Discussion

The above results demonstrate that CMRO _2_ can be measured in rats using a combination of ASE and ASL images. The method does not require administration of a gas challenge ^50, 51^, or administration of expensive isotopes ^9^. Hence this method has the potential to be a cheap, easily available method compared to gold-standard PET measurements. Little *et al.* have demonstrated similar findings using separate measures of R2 and R2* to measure OEF instead of the single measurement of R2’ from the ASE scan ^52^. In humans, qBOLD has been combined with QSM to estimate CMRO _2_ from a single multi-echo gradient-echo scan ^12^. This method shows promise, but the required modelling and processing was extremely complex. In contrast, after correction for MFGs, the ASE method only requires a fit of Equation (2) to the data. We then combined our measurement of OEF with CBF measured by ASL to generate a map of CMRO _2_ under two common anaesthetics which are known to have different effects on brain metabolism. By using a dedicated labelling coil and correcting our multi-slice 2D data with the correct post-label delay we obtained full brain maps of CBF ^39, 53^.

There were numerous technical challenges to implementing the ASE method at ultra-high field (9.4T) and the small dimensions of a preclinical system compared to previous clinical work. Foremost, MFGs were highly problematic, and adequately correcting them involved a large number of trade-offs which prevented full correction across all regions of the brain. Notably, we observed strong gradients in all three geometric directions. This required the implementation of shimming in both the slice-select (Z) and phase-encode (Y) directions. Providing an adequate number of shims required acquiring a total of 45 images per value of *τ* (nine Y-shims multiplied by 5 Z-shims), which is significantly more than the eight images that were adequate in a clinical setting ^19^. Including shim gradients in the readout direction (X) may have further reduced MFG artefacts, but at the expense of additional scan-time.

Thinner slices would also reduce the impact of the MFGs, but would also lower signal-to-noise ratio (SNR) and brain coverage. Acquiring more slices would be problematic for the ASL scan, where the maximum number is determined by the time between the end of the post-labelling time and the end of TR. Increasing TR and hence the number of slices would hence increase the ASL scan time further and lead to very different post-labelling times for different slices.

As shown in Figure 2, naïve RSS combination of the different shims leads to amplification of the Rician noise in low signal areas. We could not use the Fourier Transform approach to shim combination taken by Stone & Blockley ^19^ as the necessary reconstruction methods were not available from the manufacturer. Subtracting the average noise level from the squared magnitude images restored an adequate level of SNR. It is possible that using a method that accounts for the multi-channel nature of this data could improve the SNR further ^54, 55^.

Despite this, we found we could not reliably fit for both DBV and R2′ (data not shown). Little *et al.* used a separate Turbo Dynamic ASL scan to measure arterial blood volume, and then assumed a fixed venous/arterial blood volume ratio ^52^. As we did not acquire such a scan, we fixed DBV to a single value. Previous literature values of DBV in healthy humans vary from 1% ^56^ to 3.6% ^18^. In rodents Yablonskiy *et al.* reported a value of 3.3%, Little *et al.* reported 3.15% ^23, 52^, and Sandor *et al.* reported 3.5% (after conversion from CBV) ^57^. We opted to use the former value. Fixing DBV in this manner is not ideal as it will vary due to pathology ^52^. We hypothesise that adjusting the protocol to acquire fewer intermediate values of *τ* and additional images with high values and near *τ*=0 could improve the sensitivity to both DBV and R2’ ^58^. Increasing the maximum value of *τ* would necessitate either a corresponding increase in TE, which would reduce SNR and increase the effects of MFGs ^19^, or the use of Partial Fourier acceleration, which we found caused unacceptable blurring and intensity artefacts from the echo moving out of the EPI acquisition window ^30^. Using an alternate readout such as spiral imaging may mitigate such downsides. Such optimization was beyond the scope of the current work.

The introduction of the parameter Δ *T*, representing either the early or late arrival of the spin-echo peak, improved the stability of our fit on the edges of white matter and towards the lower front portion of the brain. We hypothesise that that these shifts were due to uncorrected MFGs, either in the X-direction causing signal to shift away from the k-space center at *τ*=0 ^30, 59^, or simply insufficient correction in the Y- and Z-directions. Further investigation of this was beyond the scope of the current work. We showed both an increase in OEF and root-mean-square error in white-matter regions, indicating that the current model does not account properly for the effects of myelin, which has a different susceptibility to other brain tissue ^60^.

Comparing our measured values of CBF, OEF and CMRO _2_ to existing literature is complicated by a wide range of measurement techniques, regions of interest, anesthetic regimes, and potential inter-species differences with humans. Starting with CBF, where the pre-clinical literature is better developed, our results appear in line with existing work. Masamoto and Kanno summarized previous literature on CBF measured by MRI and autoradiography and reported values between 102 and 247 ml/100g/min depending on the level of isoflurane anesthesia, and only 65 ml/100g/min for *α*-Chloralose measured by autoradiography ^61^. Little *et al.* reported 99–115 ml/100g/min under 1.5–2% isoflurane ^52^, while Lenz *et al.* reported 124–150 ml/100g/min depending on the level of isoflurane ^62^. We hence conclude that our CBF measurement broadly agrees with previous literature.

Previous estimates of OEF in healthy humans are 35%, measured with calibrated gas administration ^13^, and 21% using the same ASE method used here ^19^, however we note that the authors acknowledged that their choice of linear fitting deliberately underestimates OEF. Hyder *et al.* found an average grey matter OEF of 40% using PET imaging ^63^. In rats, Little *et al.* found values between 35 and 40% under Isoflurane anesthesia using an R2’ method similar to ours ^52^. He *et al.* reported mean OEFs of 23% and 38% under isoflurane and α-Chloralose respectively ^23^. This comparison to previous literature would indicate that the method presented here underestimates OEF.

Previous reported measures of CMRO _2_ in rats under α-chloralose anesthetic range include 151 ^7^, 184 ^64^, 200 ^65^, 208 ^66^ and 219 ^67^ μmol/100g/min. Our reported CMRO _2_ is approximately half that of the most commonly reported, and almost certainly due to the underestimate of OEF identified above.

Further evidence for this underestimation comes from the work of Hyder *et al.*, who measured CMRO _2 _and GU in awake humans using PET and found an Oxygen Glucose Index (OGI) of 5.3. Under pure aerobic metabolism, the stoichiometric ratio of six molecules of oxygen to one molecule of glucose would suggest an OGI of six ^68, 69^. The average OGI is equivalent to the slope of our CMRO _2_ / GU regression line, which we found to be only 2.74. Although inter-species and anesthetic effects cannot be discounted, we attribute this discrepancy to our underestimation of OEF. As discussed above, a likely cause of this underestimation is the choice of equally spread τ values, and an optimized protocol may lead to more accurate OEF estimation.

It is also possible that some of the constants chosen here may be incorrect. An obvious candidate would be the chosen value of DBV, as this roughly scales the estimate of OEF. However, decreasing the value of DBV such that our CMRO _2_ estimates agreed with literature values would require approximately halving it, which would then make the estimate of DBV itself significantly different to previous literature.

Despite this underestimation, we confirmed the expected differential effect of the anesthetics on cerebral metabolism, with close to double the rate of oxygen consumption under isoflurane than *α*-Chloralose. We note that the difference in CMRO _2_ was driven primarily by the difference in CBF which was three times higher under isoflurane, while OEF only reduced by a quarter compared to *α*-Chloralose. This is in line with the notion that mitochondria require a particular gradient of tissue oxygenation, and because less oxygen is removed from the blood during higher flow (decreased capillary transit time), it follows that OEF is decreased with increased CBF and CMRO _2_^70^.

## Conclusions

We implemented a non-invasive MRI method for measuring CMRO _2_ in rats which can be easily translated to clinical scanners. Methodological difficulties prevented measurement of DBV and we likely underestimated OEF, but future optimizations may be able to overcome these limitations. However the relative CMRO _2_ differences between anesthetics were observed, suggesting the utility of this relatively simple method for preclinical studies interested in comparing metabolic effects of treatments or pathologies.

## Data availability

### Underlying data

Figshare: CMRO _2_ in Rodents. https://doi.org/10.6084/m9.figshare.14199035.v3 ^49^.

This project contains the following underlying data:

-ROIS (ROI summary statistics in Comma Separated Value format).

-Parameter maps (mean parameter maps in Nifti format).

-Raw ASE scans (in Nifti format).

Data are available under the terms of the Creative Commons Attribution 4.0 International license (CC-BY 4.0).
