# Supplementary material for: Non-Invasive measurement of the cerebral metabolic rate of oxygen using MRI in rodents
Source: Wellcome Open Res. 2022 Aug 25;6:109. Originally published 2021 May 13. [Version 4] doi: 10.12688/wellcomeopenres.16734.4 (PMC9428501; doi:10.12688/wellcomeopenres.16734.4)
Supplement: Peer review history

# Peer review: Reviewer response for version 4

**DOI:** 10.21956/wellcomeopenres.20159.r52156

**Published:** 2022-08-30

**Authors:** Avery Berman

**Affiliations:** 1 Athinoula A. Martinos Center for Biomedical Imaging, Massachusetts General Hospital, Charlestown, MA, USA; 2 Department of Physics, Carleton University, Ottawa, ON, Canada; 3 Brain Imaging Centre, The Royal Ottawa Institute of Mental Health Research, Ottawa, ON, Canada

The authors have shown that they have thoroughly examined the issue of the shift of the R2' decay curve. My comments have been addressed.

Is the rationale for developing the new method (or application) clearly explained?

Yes

Is the description of the method technically sound?

Partly

Are the conclusions about the method and its performance adequately supported by the findings presented in the article?

Partly

If any results are presented, are all the source data underlying the results available to ensure full reproducibility?

Yes

Are sufficient details provided to allow replication of the method development and its use by others?

Partly

Reviewer Expertise:

biophysical modelling of the blood oxygenation level-dependent (BOLD) fMRI signal, high-resolution fMRI, MRI pulse sequence development

I confirm that I have read this submission and believe that I have an appropriate level of expertise to confirm that it is of an acceptable scientific standard.

# Peer review: Reviewer response for version 3

**DOI:** 10.21956/wellcomeopenres.20005.r51500

**Published:** 2022-07-27

**Authors:** Avery Berman

**Affiliations:** 1 Athinoula A. Martinos Center for Biomedical Imaging, Massachusetts General Hospital, Charlestown, MA, USA; 2 Department of Physics, Carleton University, Ottawa, ON, Canada; 3 Brain Imaging Centre, The Royal Ottawa Institute of Mental Health Research, Ottawa, ON, Canada

1. Perhaps examining the multi-channel combination on top of the shim combination is beyond the scope of the study. This could be examined in more detail in another study and the addition to the Discussion in the revised submission is appropriate.
2. Regarding the shift parameter, ΔT, its presence was surprising since it would seem to imply that the signal when τ ≠ 0 can be greater than the signal at τ = 0 (i.e., the spin-echo itself). I did not originally list any ways that the authors could try to investigate the origins of the shift because I did not want to impose one way when the authors may have preferred some other, which too could have been appropriate. Some combination of the techniques that I had in mind were (I am not asking that you perform all these suggested analyses!):
  a) Probably the most direct test would be to simulate/calculate the asymmetric spin-echo sequence (ignoring image encoding) with a range of τ offsets in a voxel with or without a static field inhomogeneity across it. Next, add various magnitudes of blipped shims. How does the “decay” curve of signal vs. τ change with shimming? Does the maximum shift from τ = 0 in any of the simulations? If you do a sum-of-squares combination of the shimmed signals, do you see a shift then? For simplicity, simulations in one-dimension, with a linear gradient inhomogeneity, without diffusion and completely ignoring any effect of CBV/dHb should suffice to isolate the effect of shimming/macroscopic field inhomogeneity.
  b) Alternatively, using the data on hand, consider voxels that showed a large shift parameter and compare them to voxels that had a negligible shift parameter in some of the following ways: Look at the entire “decay” curve of signal vs. τ without shimming. Is the maximum shifted from τ = 0? How do these decay curves look at various shim levels? Does the maximum shift? What happens to the signal at τ = 0 with shimming? Does the signal for τ ≠ 0 actually exceed the signal at τ = 0 (unshimmed) for some shimming value?
  c) Again using the experimental data, is the shift something like a fitting artifact? For voxels with large ΔT, are the decay curves vs. τ steep or shallow (relative to the peak intensity at ΔT)? If shallow, i.e., R2' is small, then could the peak shift be the result of trying to fit a peak to a relatively flat curve + noise? Is there some correlation between R2' and ΔT that may support this?

**Minor Comment:**

 In the Imaging Protocol subsection of the Methods, the authors state, “For the ASE sequence we modified the manufacturer’s spin-echo EPI sequence to allow the 180° refocusing pulse to be offset by τ as defined above.” Is the refocusing pulse offset by τ or τ/2 (resulting in shifts of the echo of 2τ or τ, respectively)?

Is the rationale for developing the new method (or application) clearly explained?

Yes

Is the description of the method technically sound?

Partly

Are the conclusions about the method and its performance adequately supported by the findings presented in the article?

Partly

If any results are presented, are all the source data underlying the results available to ensure full reproducibility?

Yes

Are sufficient details provided to allow replication of the method development and its use by others?

Partly

Reviewer Expertise:

biophysical modelling of the blood oxygenation level-dependent (BOLD) fMRI signal, high-resolution fMRI, MRI pulse sequence development

I confirm that I have read this submission and believe that I have an appropriate level of expertise to confirm that it is of an acceptable scientific standard, however I have significant reservations, as outlined above.

# Response

**Published:** 2022-08-04

**Authors:** Tobias C. Wood

**Affiliations:** King's College London, UK

We thank the reviewer for their further comments.

 Minor Comment: The shift of tau/2 is correctly defined in the Theory section. The manuscript has been edited to make this clearer.

 Point 2: The acquired signal can be greater than at the spin echo in the presence of Macroscopic Field Gradients. This is the reason Z-shimming was proposed for OEF mapping by Blockley et al. Investigations as proposed in (b) were performed during study setup and led to the idea of adding the shift parameter. There are no obvious correlations as suggested in (c), which can be seen visually in figure 4 (the shift parameter has a different spatial pattern to R2’). The decay curves are indeed fairly flat and noisy, we have already included discussion of the trade-offs that would be involved in better estimation of R2’ - “We hypothesise that adjusting the protocol to acquire fewer intermediate values of *τ*and additional images with high values and near *τ*=0 could improve the sensitivity to both DBV and R2’ ^58^. Increasing the maximum value of *τ* would necessitate either a corresponding increase in TE, which would reduce SNR and increase the effects of MFGs ^19^, or the use of Partial Fourier acceleration, which we found caused unacceptable blurring and intensity artefacts from the echo moving out of the EPI acquisition window ^30^”. The investigations suggested in (a) would be significant extra work that is beyond the scope of this manuscript. We have investigated the fitting of our data as thoroughly as we consider reasonable, and further investigations are beyond the scope of this work. We have now added the raw data to the downloadable data, which will allow interested parties to conduct additional investigations if they so choose.

# Peer review: Reviewer response for version 2

**DOI:** 10.21956/wellcomeopenres.18968.r45804

**Published:** 2022-01-24

**Authors:** Yolanda Ohene

**Affiliations:** 1 The University of Manchester, Manchester, UK

No further comments.

Is the rationale for developing the new method (or application) clearly explained?

Yes

Is the description of the method technically sound?

Yes

Are the conclusions about the method and its performance adequately supported by the findings presented in the article?

Yes

If any results are presented, are all the source data underlying the results available to ensure full reproducibility?

Yes

Are sufficient details provided to allow replication of the method development and its use by others?

Yes

Reviewer Expertise:

MRI - Arterial Spin Labelling

I confirm that I have read this submission and believe that I have an appropriate level of expertise to confirm that it is of an acceptable scientific standard.

# Peer review: Reviewer response for version 2

**DOI:** 10.21956/wellcomeopenres.18968.r45803

**Published:** 2021-10-11

**Authors:** Avery Berman

**Affiliations:** 1 Athinoula A. Martinos Center for Biomedical Imaging, Massachusetts General Hospital, Charlestown, MA, USA; 2 Department of Physics, Carleton University, Ottawa, ON, Canada; 3 Brain Imaging Centre, The Royal Ottawa Institute of Mental Health Research, Ottawa, ON, Canada

Wood *et al. *have updated their manuscript to help address most of my previous concerns and those of Reviewer 1. I would like to thank the authors for adding some key references from the literature. I still have reservations for my original comments 1 and 2.

 The authors have referenced the paper by Miller and Joseph (1993) regarding the validity of the noise floor correction on relaxometry (my original comment 1). The noise floor correction from Miller and Joseph was specifically for the root-sum-of-squares (RSS) combination of single-channel real and imaginary data. Here, the authors have combined images from multiple z-shims (>2) which themselves are the result of RSS combination of 4-channel data. A different correction factor may be required for 4-channel data, although it’s not immediately obvious to me how this then applies to the combination of multiple z-shimmed images (perhaps they can be treated as additional channels). Koay and Basser (2006, J Magn Reson) ^1^ have examined this correction factor for multi-channel RSS (see also a follow-up paper from Bai *et al.* [2014, J Magn Reson]). ^2^

 Regarding the *R*_2_' fitting parameter ΔT, which was introduced in the original manuscript to account for shifts in the signal maximum away from the nominal spin-echo image at τ = 0. This was comment 2 in my previous review. In their response, the authors have suggested that this could be due to large macroscopic field inhomogeneity along the non-shimmed direction (x-axis). I am still not convinced that this is the source of the shift since the whole point of the refocusing pulse is to refocus dephasing that arises from these static field inhomogeneities at the spin-echo. The reference that was added from Chen et al., was specifically discussing gradient-echo, and its relevance to asymmetric spin-echo was mentioned; however, their theory does not apply to the spin-echo. I think there are multiple ways one could go about investigating the nature of this shift with the data on hand or with some straightforward modelling. This may not, in the end, affect the fitted *R*_2_' values, but I think it would go a long way to help solidify the methodological underpinnings of the proposed *R*_2_'/OEF mapping.

Is the rationale for developing the new method (or application) clearly explained?

Yes

Is the description of the method technically sound?

Partly

Are the conclusions about the method and its performance adequately supported by the findings presented in the article?

Partly

If any results are presented, are all the source data underlying the results available to ensure full reproducibility?

Yes

Are sufficient details provided to allow replication of the method development and its use by others?

Partly

Reviewer Expertise:

biophysical modelling of the blood oxygenation level-dependent (BOLD) fMRI signal, high-resolution fMRI, MRI pulse sequence development

I confirm that I have read this submission and believe that I have an appropriate level of expertise to confirm that it is of an acceptable scientific standard, however I have significant reservations, as outlined above.

## References

[1] : Analytically exact correction scheme for signal extraction from noisy magnitude MR signals. *J Magn Reson* .2006;179(2) : 10.1016/j.jmr.2006.01.016 317-22 10.1016/j.jmr.2006.01.016 16488635

[2] : A framework for accurate determination of the T₂ distribution from multiple echo magnitude MRI images. *J Magn Reson* .2014;244: 10.1016/j.jmr.2014.04.016 53-63 10.1016/j.jmr.2014.04.016 24859198PMC4086921

# Response

**Published:** 2021-12-08

**Authors:** Tobias C. Wood

**Affiliations:** King's College London, UK

We thank the reviewer for their further comments.

1. The reviewer did not raise the issue of the multi-channel combination or provide these references in their first review. This topic is far beyond the scope of the paper. The principal reason for the noise correction was to remove background noise, as described in the caption of figure 3, such that processing steps such as brain extraction and registration could be achieved. The low echo-offset that we could achieve means that within the brain there is not a large amount of signal decay, i.e. within the brain we appear to be operating in a reasonable SNR regime, and we do not reach the noise floor of T2' decay.
2. The introduction of the shift parameter was the most straightforward, empirical way to address the issue. As can be seen in figure 4 the resulting value of the shift parameter is close to zero over most of the brain including the cortex. It is hence not the source of the R2' prime underestimation we observed, which occurred over the whole brain, and is by far the more important issue which we have now dedicated significant space to discussing. The reviewer suggests in his reply that there are multiple ways to investigate the nature of the T2' shift but a list of such ways is missing, hence it is not possible to respond further to this comment.

# Peer review: Reviewer response for version 1

**DOI:** 10.21956/wellcomeopenres.18455.r43881

**Published:** 2021-06-04

**Authors:** Yolanda Ohene

**Affiliations:** 1 The University of Manchester, Manchester, UK

Wood *et al*. have combined two techniques, arterial spin labelling (ASL) and asymmetric spin-echo (ASE), that capture the cerebral blood flow (CBF) and the oxygen extraction fraction (OEF), respectively, to provide a non-invasive measurement of cerebral metabolic rate of oxygen (CMRO _2_).

 CMRO _2 _measurements were taken in rodents under two different anaesthetic conditions. The authors demonstrate that CMRO _2_ is lower under α-Chloralose anaesthesia compared to isoflurane anaesthesia. The metabolic activity was validated by measuring glucose consumption using autoradiography under the same two anaesthetic conditions. The glucose metabolic rate is also lower under α-Chloralose anaesthesia. The study design resulted in a clear separation of the OEF, CBF, CMRO _2_ and glucose utilisation (GU) under the two anaesthetic conditions, in almost all brain regions, demonstrating the successful implementation of this non-invasive method.

 Minor comments:

- Could the authors comment on the effect of fixing the deoxygenated blood volume (DBV) in the OEF measurements, particularly considering that there is a significant difference in the CBF between the two anaesthesia groups which may have an influence on the DBV value?
- Introduction, Paragraph 1: Typo – “Bloody” to "Blood".
- Figure 5: Labelling of the coloured bars are overlapping on the bottom row images.

Is the rationale for developing the new method (or application) clearly explained?

Yes

Is the description of the method technically sound?

Yes

Are the conclusions about the method and its performance adequately supported by the findings presented in the article?

Yes

If any results are presented, are all the source data underlying the results available to ensure full reproducibility?

Yes

Are sufficient details provided to allow replication of the method development and its use by others?

Yes

Reviewer Expertise:

MRI - Arterial Spin Labelling

I confirm that I have read this submission and believe that I have an appropriate level of expertise to confirm that it is of an acceptable scientific standard.

# Response

**Published:** 2021-10-22

**Authors:** Tobias C. Wood

**Affiliations:** King's College London, UK

We thank the reviewer for their further comments.

1. The reviewer did not raise the issue of the multi-channel combination or provide these references in their first review. This topic is far beyond the scope of the paper. The principal reason for the noise correction was to remove background noise, as described in the caption of figure 3, such that processing steps such as brain extraction and registration could be achieved. The low echo-offset that we could achieve means that within the brain there is not a large amount of signal decay, i.e. within the brain we appear to be operating in a reasonable SNR regime, and we do not reach the noise floor of T2' decay.
2. The introduction of the shift parameter was the most straightforward, empirical way to address the issue. As can be seen in figure 4 the resulting value of the shift parameter is close to zero over most of the brain including the cortex. It is hence not the source of the R2' prime underestimation we observed, which occurred over the whole brain, and is by far the more important issue which we have now dedicated significant space to discussing. The reviewer suggests in his reply that there are multiple ways to investigate the nature of the T2' shift but a list of such ways is missing, hence it is not possible to respond further to this comment.

# Peer review: Reviewer response for version 1

**DOI:** 10.21956/wellcomeopenres.18455.r43877

**Published:** 2021-06-02

**Authors:** Avery Berman

**Affiliations:** 1 Athinoula A. Martinos Center for Biomedical Imaging, Massachusetts General Hospital, Charlestown, MA, USA; 2 Department of Physics, Carleton University, Ottawa, ON, Canada; 3 Brain Imaging Centre, The Royal Ottawa Institute of Mental Health Research, Ottawa, ON, Canada

Wood *et al. *have presented a new *R*_2_'-based method for quantifying the oxygen extraction fraction (OEF) and the cerebral metabolic rate of O _2_ (CMRO _2_) in rats. The novelty here lies in i) the variable shimming approach used to reduce the effect of macroscopic field inhomogeneities that otherwise contaminate the contribution to *R*_2_' from deoxyhemoglobin in the vasculature, ii) the application of this approach to compare various brain physiological parameters (OEF, CBF, CMRO _2_) under two different anesthetics (isoflurane and alpha-Chloralose) and iii) the comparison of CMRO _2_ to the glucose uptake (GU) by autoradiography.

 The study shows large differences in brain physiology under the different anesthetics, consistent with previous findings. The final finding that the authors fit a CMRO _2_ to GU ratio of 6.4 is quite remarkable, considering the theoretical ratio is 6 (i.e., 6 molecules of O _2_ consumed per glucose during oxidative glycolysis). I do have significant concerns, however, with the quantification of *R*_2_' given that multiple previously unpublished techniques were employed that I think would benefit from further validation. Given that the OEF and CMRO _2_ quantification rely on the estimate of *R*_2_', this does have important consequences for the comparisons of the absolute OEF and CMRO _2_ values across the two anesthetics and for the absolute CMRO _2_:GU ratio.

1. To the best of my knowledge, the way the shimmed images were combined through root sum of squares is a previously unpublished approach. Similarly, the proposed noise floor correction via subtraction of the mean-squared noise signal in the background is also novel to this study. While the noise subtraction does appear to improve the contrast-to-noise, it is not apparent that the desired absolute signal levels are preserved. Since these images are later used for absolute quantification of *R*_2_', it is important that the signal levels are not systematically biased, but if they are, at least that bias can be characterized. It would be reassuring to see a validation of these new techniques.
2. The authors introduced the *R*_2_' fitting parameter ΔT to account for shifts in the signal maximum away from the nominal spin-echo time at τ = 0. The magnitude of the shifts, ±25 ms, are dramatic and are previously unheard of (to the best of my knowledge). This is possibly related to the shimming and/or the shim image combination. Again, some form of external validation of this parameter to better understand its origin would help give better trust in the resulting *R*_2_' fits.
3. Another major limitation of this study was that the *R*_2_' fitting required fixing the value of deoxygenated blood volume (DBV) to 3.3%. While this may have helped stabilize the fit, it would lead to OEF estimates that would differ from their true values depending on how the true DBV compared to the assumed value, and this points to issues with the model and/or data. As DBV is regionally varying, this limits the validity of the OEF estimates. Could the authors elaborate on how they performed the fitting? In Eq. 2, there is the quadratic decay period for tau < Tc and the linear decay period for tau > Tc. Also note, typically studies have used a factor of 1.5*Tc for the transition period, although this would depend on how the authors have defined Tc (e.g, refs. 17 or 19). Was data from all periods fit? How was Tc determined without a priori knowing *R*_2_'? In previous quantitative BOLD studies, the linear decay period is extrapolated to the signal at tau = 0 and the difference between this signal and the measured signal is proportional to DBV, was this fitting approach used here?

**Minor comments:**

1. In the opening paragraph, the authors refer to “quantitative BOLD” using calibration with gases. This calibration with gases is referred to as “calibrated” BOLD or fMRI, not quantitative BOLD. Quantitative BOLD is a gas-free technique which the methods in this manuscript are based on.
2. The values for the arterial O _2_ concentration, Ca, are based on human physiological parameters. The authors should consider using values for rats. See, e.g., Gagnon *et al.*, J Neurosci (2015). ^1^
3. The labels are cropped in Figure 5
4. In the middle of the Discussion section, the authors compare their OEF estimates to He *et al.*’s where they state findings that were in line with the findings of this manuscript. I think that stating the results being in line with each other is a bit generous as the OEF values in the manuscript differ by 35% (iso.) and 44% (alpha-chlor.) relative to those from He et al.
5. It would be nice to see a discussion of other contributions to *R*_2_' contamination beyond macroscopic field inhomogeneities, such as iron depositions or regionally varying myelination since these may also bias the OEF estimate and would be important to be aware of in (pre)clinical studies.
6. It would be helpful to label some of the key ROIs discussed in the manuscript, in particular the inferior colliculus.

Is the rationale for developing the new method (or application) clearly explained?

Yes

Is the description of the method technically sound?

Partly

Are the conclusions about the method and its performance adequately supported by the findings presented in the article?

Partly

If any results are presented, are all the source data underlying the results available to ensure full reproducibility?

Yes

Are sufficient details provided to allow replication of the method development and its use by others?

Partly

Reviewer Expertise:

biophysical modelling of the blood oxygenation level-dependent (BOLD) fMRI signal, high-resolution fMRI, MRI pulse sequence development

I confirm that I have read this submission and believe that I have an appropriate level of expertise to confirm that it is of an acceptable scientific standard, however I have significant reservations, as outlined above.

## References

[1] : Quantifying the microvascular origin of BOLD-fMRI from first principles with two-photon microscopy and an oxygen-sensitive nanoprobe. *J Neurosci* .2015;35(8) : 10.1523/JNEUROSCI.3555-14.2015 3663-75 10.1523/JNEUROSCI.3555-14.2015 25716864PMC4339366

# Response

**Published:** 2021-09-09

**Authors:** Tobias C. Wood

**Affiliations:** King's College London, UK

We thank the reviewer for their constructive feedback. Responses to the individual points are below.

 Major:

  

1. A key reference describing the use of the root-sum-squares method for combining z-shimmed images (Spencer & Constable 1999) was omitted. This has now been corrected. Similarly, the noise floor correction method has also been previously published (Miller & Joseph 1993, reference 40) where it was used in the context of T2/R2 fitting. While these techniques are old to our knowledge they have not been invalidated.
2. We agree with the reviewer that such shifts have not been described previously in the literature, however the linear extrapolation method used by Blockley et al cannot detect such shifts even if they are present. In addition, previous literature in this area has used human subjects and lower field strengths. We suspect that strong MFGs in the x-direction, which we could not compensate due to scan time constraints, may be the underlying cause of such shifts. This putative explanation has been added to the discussion, along with an additional reference (Chen et al)
3. We agree that fixing the value of DBV is a limitation of this study. We previously used a value of 1.5Tc for the transition period and Tc=R2’/DBV and a non-linear fit across all data points. Whave now updated the code (and relevant equations in the manuscript) to use the full integral form of Yablonskiy et al, but note that this made very little difference to the fitted values compared to the asymptotic form of the equations. This code is now available in version 3.3 of our toolbox.  Likely, a major contributor to the difficulty in fitting for DBV is the relatively small maximum value of τ we could achieve within imaging constraints and only having a single τ=0 image. A more efficient protocol may be to acquire multiple τ=0 images and fewer intermediate values of τ. This point has been expanded in the discussion.

Minor comments:

1. Thank you for the clarification, the introduction has been reworded accordingly.
2. We thank the reviewer for bringing this to our attention. Please see the notes on the amendments at the start of the text for a full discussion of this issue.
3. The labels have been corrected in figure 5.
4. On reflection we agree with the reviewer. The sentence has been amended to state that the values in He et al are further evidence that our method currently underestimates OEF.
5. Discussion of the effect of myelination has been added, as it is clear the current model does not account for this.
6. Due to the 3D nature of the ROIs, displaying all of them would require an additional figure. The Inferior Colliculus has been marked on an existing figure with arrows.
